# The feasibility and efficiency for constructing arteriovenous fistula with <2 mm vein—a systematic review and meta-analysis

**DOI:** 10.3389/fcvm.2023.1226136

**Published:** 2023-09-18

**Authors:** Ruijia Feng, Siwen Wang, Jianwen Yu, Xunhua Zheng, Wei Chen, Xin Wang, Guangqi Chang

**Affiliations:** ^1^ Department of Vascular Surgery, National-Guangdong Joint Engineering Laboratory for Diagnosis and Treatment of Vascular Diseases, The First Affiliated Hospital of Sun Yat-sen University, Guangzhou, China; ^2^ Department of Nephrology, NHC Key Laboratory of Nephrology, Guangdong Provincial Key Laboratory of Nephrology, The First Affiliated Hospital of Sun Yat-sen University, Guangzhou, China

**Keywords:** small-caliber vein, arteriovenous fistula, functional maturation rate, end-stage renal disease, feasibility

## Abstract

**Background:**

Autogenous arteriovenous fistula (AVF) is an efficient hemodialysis access for patients with end-stage kidney disease (ESKD). The specific threshold of vein diameter still not reached a consensus.

**Method:**

We conducted a comprehensive search in PubMed, Embase, and Web of Science databases for articles which comparing the treatment outcomes of AVF with 2 mm as vein diameter threshold. Fixed and random effect model were used for synthesis of results. Subgroup analysis was designed to assess the risk of bias.

**Result:**

Eight high-quality articles were included finally. Among a total of 1,075 patients (675 males and 400 females), 227 and 809 patients possessed <2 mm and ≥2 mm vein respectively. Apart from gender and coronary artery disease (*P* < 0.05), there was no significant difference in age, diabetes, hypertension or radial artery between maturation and non-maturation groups. The functional maturation rate was lower in patients with <2 mm vein according to fixed effect model [OR = 0.19, 95% CI (0.12, 0.30), *P* < 0.01]. There was no significant difference in primary [OR = 0.63, 95% CI (0.12, 3.25), *P* = 0.58] or cumulative patency rates [OR = 0.40, 95% CI (0.13, 1.19), *P* = 0.10].

**Conclusion:**

Vein diameter less than 2 mm has a negative impact on the functional maturation rate of AVF, while it does not affect the primary and cumulative patency rates (12 months).

## Introduction

Hemodialysis is one of the most essential treatment strategies for patients with end-stage kidney disease (ESKD) (1). Traditionally, central venous catheters (CVC) were constructed for patients for long time regular hemodialysis. However, the incidence of thrombosis and infection were relatively high and the nursing work was also complicated, which led to development of alternative hemodialysis access used for maintenance hemodialysis (2, 3). The National Kidney Foundation's Kidney Disease Outcomes Quality Initiative (KDOQI) had recommended that autogenous arteriovenous fistula (AVF) was most preferred, followed by synthetic graft (AVG) and CVC in hemodialysis patients (4). The maturation rate of AVFs varied in a wide range in different studies and the previous systematic review estimated the pooled failure rate was 23% (5). The vascular condition, especially the vein diameter was one of the most important influencing factors (6, 7). We usually use vein greater than 2 mm for AVF in clinic, and the patency rate and maturation rate were satisfactory, which had reached consensus among vascular surgeons. However, vein lesser than 2 mm is not a clearly contraindication for surgery, which required careful preoperative vascular examination and postoperative maintenance and follow-up (4). The aim of this study was to compare the long-term outcomes of AVF with different diameter veins, and we expected to broaden the range of suitable vein diameters to provide more opportunities for ESKD patients with vein lesser than 2 mm.

## Methods

### Literature search and inclusion

We conducted a thorough search in the PubMed, Embase, and Web of Science databases for all relevant articles until March 1st, 2023. The key words included “arteriovenous fistula”, “vein” and “diameter”. After removing duplicated articles, additional items were sought by manual review the reference of all articles. We initially screened the articles mainly by the title and abstract, then we read the full text of all potentially eligible articles carefully. The inclusion criteria were listed as follows: (1) articles were written in English; (2) study design was randomized controlled trial (RCTs) or observational study; (3) the AVF was constructed for hemodialysis in renal failure patients; (4) the AVF was constructed by autogenous veins;(5) the patients with different vein diameter were compared on outcome indicators such as functional maturation rate or postoperative patency rates; (6) the threshold for patients grouping was 2 mm on vein diameter. The exclusion criteria included: (1) no independent record of functional maturation in patients with different vein diameter; (2) difficult to make a distinction between AVF and AVG. The whole review process was accomplished by two authors independently and the controversies were discussed strictly to achieve consensus finally. The study was designed and reported in accordance with Preferred Reporting Items for Systematic Review and Meta-Analysis (PRISMA) Statements (8) and guidelines and recommendations of Meta-analysis of Observational Studies in Epidemiology (MOOSE) (9) respectively. Patients or the public were not involved in the design, or conduct, or reporting, or dissemination plans of our research.

### Data extraction and definition

A predefined data extraction sheet was used to record all study characteristics from all included articles: title, first author, year of publication, country, study design and number of patients in different groups. Several baseline characteristics and procedure outcomes including age, gender, previous history, follow-up time, surgical procedure, vein diameter, tourniquet using, functional maturation rate, primary patency rate, cumulative patency rate and so on were also recorded for further comparison. The data was recorded by two authors with repeated confirmation. If the relevant indicators above were not clearly illustrated or not mentioned at all in the article, we contacted the first and corresponding authors to ensure the integrity and accuracy of the data. According to the KDOQI clinical practice guideline for vascular access: 2019 update, the functional maturation was defined as a fistula became suitable for providing prescribed dialysis consistently with 2 needles. The duration of time from fistula placement to thrombosis or any intervention to facilitate, maintain, or re-establish patency (e.g., angioplasty) was defined as the primary patency, while the duration of time from fistula placement to access abandonment was defined as cumulative patency (same as secondary patency).

### Quality assessment

We used the Newcastle–Ottawa scale (NOS) (10) for cohort study to evaluate the methodological quality of the included articles. The total NOS score ranged from 0 to 9, which was divided into three parts—participants selection, comparability and outcome. Two authors independently assessed the articles and mean scores were calculated. Cohort studies were graded as follows: 0–3 = low, 4–6 = moderate, and 7–9 = high (11). In addition, the certainty of evidence was assessed in accordance with Grading of Recommendations Assessment, Development, and Evaluation (GRADE) system (12) on GRADEpro website (https://www.gradepro.org/).

### Statistical analysis

Categorical variables were presented by dividing the number of events by the number of cases. The bilateral *χ*^2^ test or Fisher's exact test was used for comparison. The continuous variables were shown as the mean ± standard deviation (SD) or median and range (quartile) and the *t*-test or Mann–Whitney *U*-test was used. Pooled odds ratio (OR) and 95% confidence intervals (CIs) were showed in forest plot. The heterogeneity was calculated by *I*^2^, which represented the proportion of the difference caused by non-sampling error in the total heterogeneity (13). *I*^2 ^< 50% indicated low heterogeneity and the fixed effect model was the best choice while *I*^2 ^≥ 50% indicated high heterogeneity and the random effect model was more appropriate (14). To clarify the potential source of heterogeneity, we performed sensitivity analysis by leave one-out approach to find studies design bias and confounding factors. The publication bias was evaluated by Egger's test and funnel plot. Subgroup analysis was conducted by gender, tourniquet using and vein diameter distribution based on fix effect model (15). A *P*-value < 0.05 was considered statistically significant. All statistical analysis was conducted in R studio (version 4.2.0; https://www.r-project.org) with “meta” and “metafor” R packages.

## Results

### Study selection and evaluation

**Figure 1:**
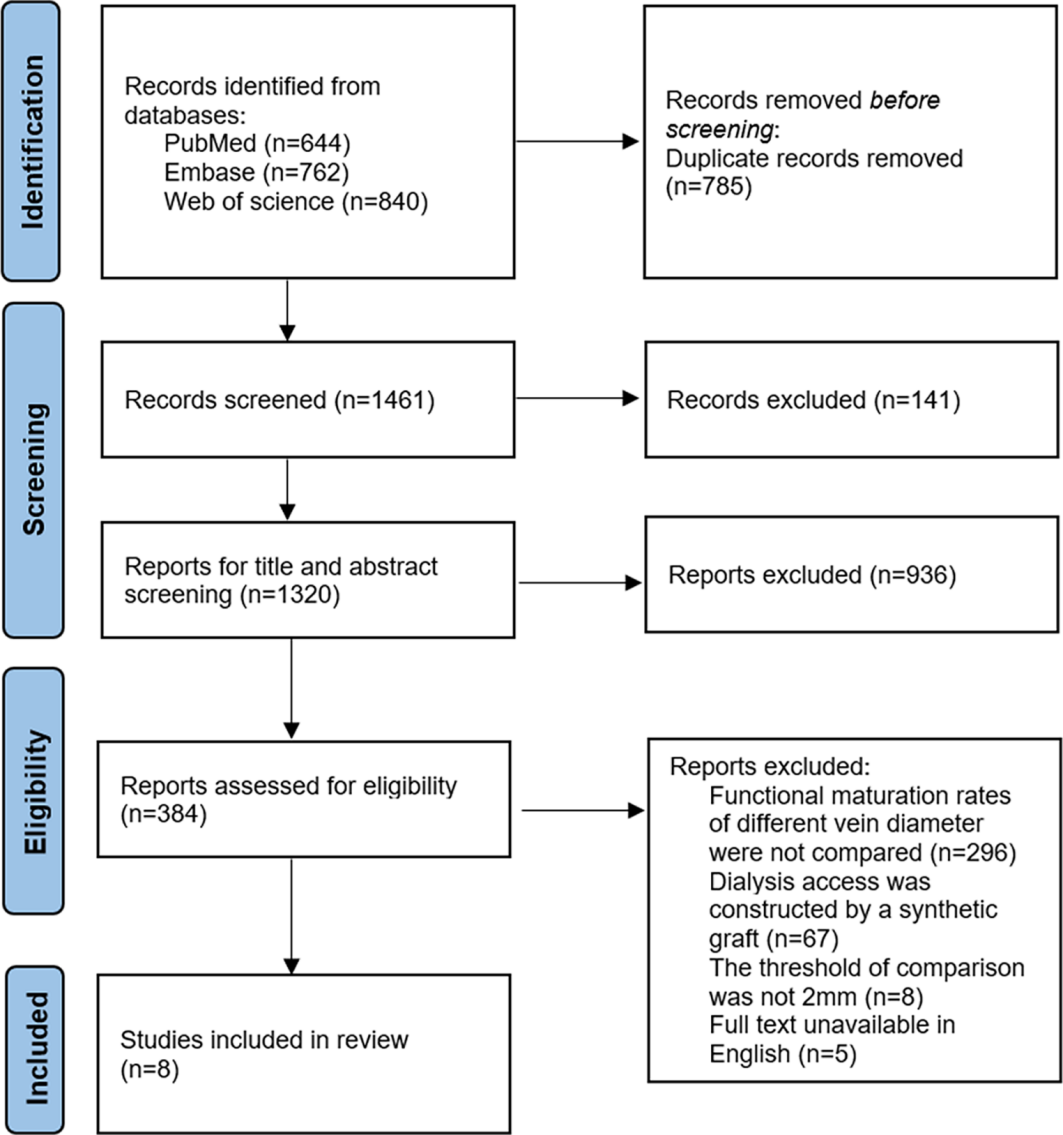
Preferred reporting items for systematic review and meta-analysis (PRISMA) flow diagram of study screening and inclusion process.

The article screening process is presented in Figure 1. Two thousand two hundred forty-six articles were searched firstly (644 from PubMed, 762 from Embase and 840 from Web of science) and 1,320 articles progressed to further review after removing duplicated records. 384 articles were preliminarily included after title and abstract screening. Two hundred ninety-six articles were excluded for lacking comparison on functional maturation rates between different vein diameters, 67 for involving synthetic graft, 8 for unexpected diameter threshold and 5 for non-English articles. Finally, 8 articles were included in this systematic review and meta-analysis (16–23). The basic characteristic of all included articles was showed in Table 1. All articles were cohort studies. Three of them were prospective study (16, 17, 19) while others were designed retrospectively. Among a total of 1,075 patients (675 males and 400 females), 227 and 809 patients had available ultrasound data and possessed <2 mm and ≥2 mm vein respectively, while others did not provide available ultrasound data. Among these, most of cases were constructed with radio-cephalic AVF except 11 cases with brachial-cephalic AVF and 5 cases with brachial-basilic AVF. Based on NOS evaluation, 6 of 8 included articles were assessed as high quality and 4 of them reached the maximum score of 9 (18, 20–23) (Supplementary Table S1).

**Table 1:** A brief summary of 8 included studies.

Study	Country	Study design	Patients	Sex		Tourniquet	Vein diameter		Follow-up time (months)	Mean age (years)
				Male	Female		<2 mm	≥2 mm		
Wong et al., (16)	UK	prospective	60	37	23	Yes	5	49	3	58.43
Mendes et al., (17)	USA	prospective	44	35	9	No	19	25	3	55.5
Park et al., (18)	Korea	retrospective	186	116	70	Yes	58	128	45.8 ± 17.0	56.86
Hörer et al., (19)	Sweden	prospective	31	17	14	No	12	19	12	63
Wilmink et al., (20)	UK	retrospective	507	329	178	Yes	8	499	12	NA
Hou et al., (22)	China	retrospective	88	53	35	No	21	49	12	54.11
Hussain et al., (21)	Pakistan	retrospective	38	13	25	Yes	34	4	3	46.76
Feng et al., (23)	China	retrospective	121	75	46	Yes	70	36	12	53.88

NA: not available.

### Outcome comparison

**Figure 2:**
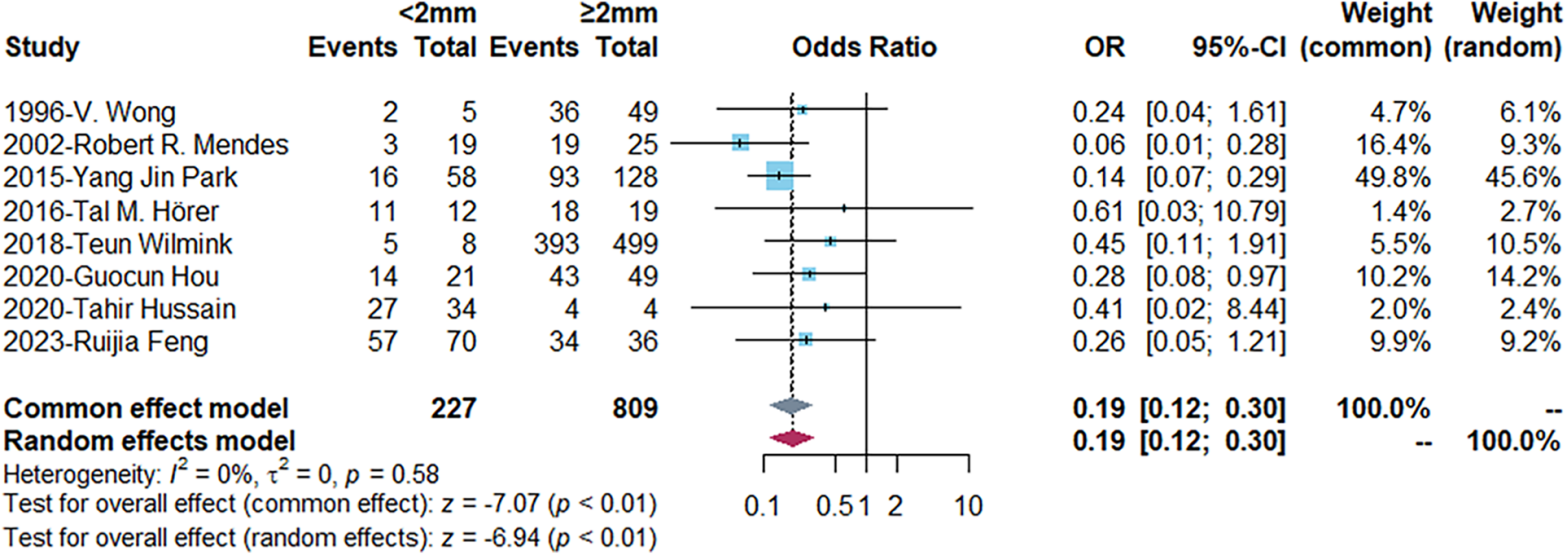
Forest plots demonstrating the comparison of functional maturation rates in different vein diameter groups. Events, number of fistulas with functional maturation; OR, odds ratio; CI, confidence interval. The vertical line represents an odds ratio of 1. For each study, the blue box represents the ratio of the occurrence of its outcome event, and the horizontal line represents the confidence interval for this ratio. The leftmost vertical dotted line represents the final ratio based on the weighting of the different studies. *I*-square represented heterogeneity between eligible articles.

**Figure 3:**
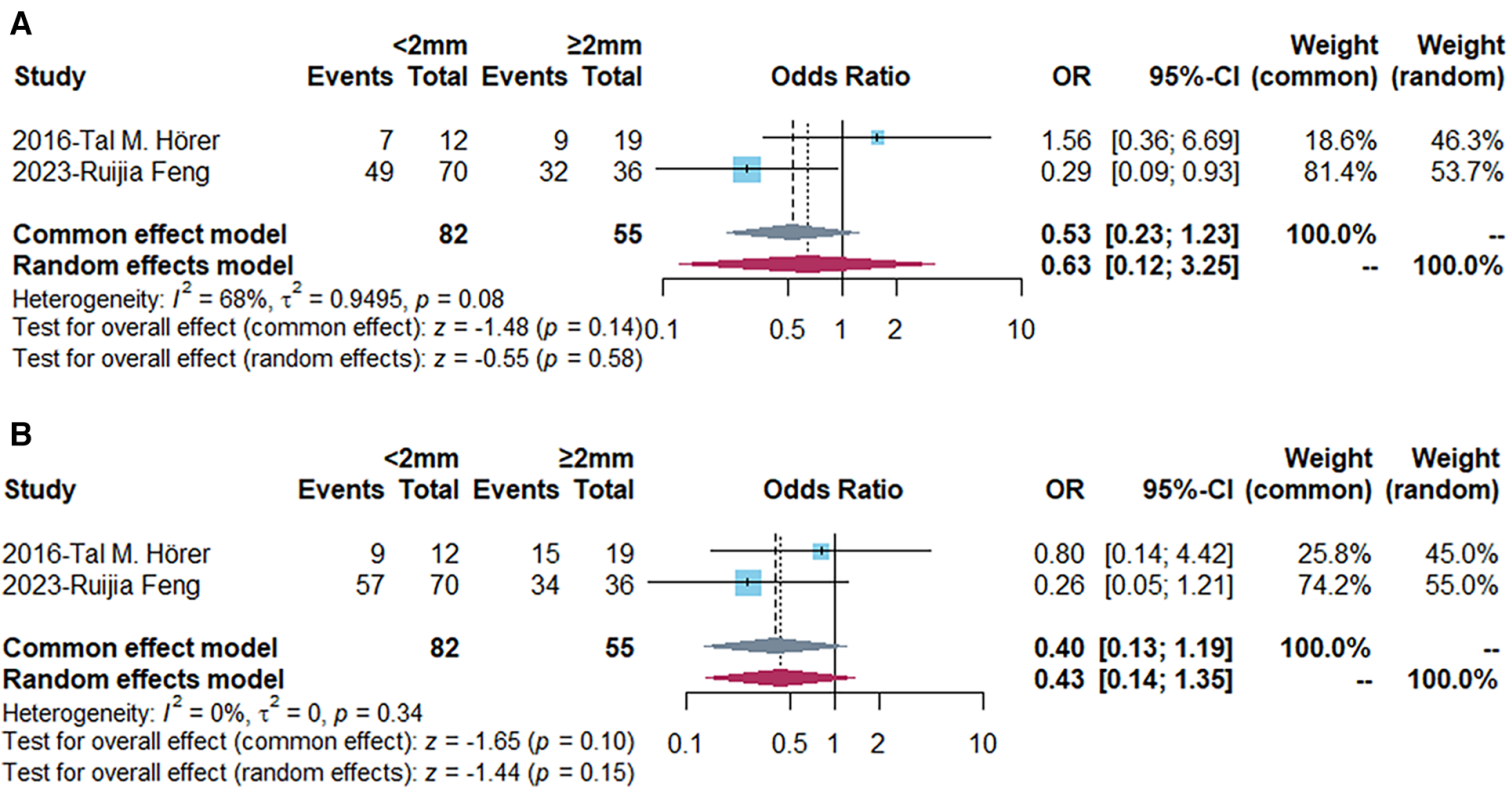
Forest plots demonstrating the comparison of primary (**A**) and cumulative (**B**) patency rates (12 months) in different vein diameter groups. Events, number of fistulas with functional maturation; OR, odds ratio; CI, confidence interval. The vertical line represents an odds ratio of 1. For each study, the blue box represents the ratio of the occurrence of its outcome event, and the horizontal line represents the confidence interval for this ratio. The leftmost vertical dotted line represents the final ratio based on the weighting of the different studies. *I*-square represented heterogeneity between eligible articles.

To explore the influencing factors of AVF maturation, we compared some baseline indicators firstly (Table 2). Apart from gender and coronary artery disease, there was no significant difference in age, diabetes, hypertension or radial artery diameter between maturation and non-maturation groups. The functional maturation rate of AVF was higher in males and lower in coronary artery disease patients (*P* < 0.05). The functional maturation rates for patients with different vein diameter were demonstrated in Table 3. The forest plot indicated that the functional maturation rate was lower in patients with <2 mm vein according to fixed effect model [OR = 0.19, 95% CI (0.12, 0.30), *P* < 0.01] (Figure 2). There was no significant difference in one-year primary [OR = 0.63, 95% CI (0.12, 3.25), *P* = 0.58] or one-year cumulative patency rates [OR = 0.40, 95% CI (0.13, 1.19), *P* = 0.10] (Figure 3).

**Table 2:** Comparison of the baseline characteristic in 5 available studies.

	Park et al., (18)		Wilmink et al., (20)		Hou et al., (22)		Hussain et al., (21)		Feng et al., (23)		Total		*P*-value
	Maturation	Non-maturation	Maturation	Non-maturation	Maturation	Non-maturation	Maturation	Non-maturation	Maturation	Non-maturation	Maturation	Non-maturation	
Patients	164	22	398	109	67	21	31	6	91	15	751	173	
Age	57 ± 14.1	55.8 ± 15.2	NA	NA	53.51 ± 14.46	55.14 ± 12.52	NA	NA	51.75 ± 15.15	59.67 ± 18.29	54.79 ± 14.62	56.56 ± 15.03	0.399
Male	102	14	282	47	40	13	10	3	56	8	490	85	<0.001*
Female	62	8	116	62	27	8	21	3	35	7	261	88	
Diabetes	88	16	173	45	NA	NA	23	5	28	7	312	73	0.653
Hypertension	131	16	NA	NA	NA	NA	20	1	84	15	235	32	0.316
Coronary artery disease	23	6	NA	NA	NA	NA	NA	NA	7	6	30	12	0.002*
Diameter of radial artery	2.70 ± 0.60	2.50 ± 1.00	NA	NA	2.26 ± 0.35	2.20 ± 0.39	NA	NA	2.16 ± 0.55	2.19 ± 0.50	2.46 ± 0.60	2.31 ± 0.71	0.090
Radial artery <2 mm	NA	NA	15	12	NA	NA	NA	NA	29	3	44	15	0.382
Radial artery ≥2 mm	NA	NA	383	97	NA	NA	NA	NA	62	12	445	109	

NA: not available.

* Significant difference.

**Table 3:** Functional maturation rate for patients with different vein diameter.

	Vein diameter <2 mm			Vein diameter ≥2 mm		
	Total	Events	Rate	Total	Events	Rate
Without tourniquet						
Mendes et al., (17)	19	3	15.8%	25	19	76.0%
Hörer et al., (19)	12	11	91.7%	19	18	94.7%
Hou et al., (22)	21	14	66.7%	49	43	87.8%
With tourniquet						
Wong et al., (16)	5	2	40.0%	49	36	73.5%
Park et al., (18)	58	16	27.6%	128	93	72.7%
Wilmink et al., (20)	8	5	62.5%	499	393	78.8%
Hussain et al., (21)	34	27	79.4%	4	4	100.0%
Feng et al., (23)	70	57	81.4%	36	34	94.4%

### Risk of bias

As for the subgroup analysis, 5 articles evaluated the vein diameter with tourniquet by ultrasound and 7 articles enrolled more males. Considering the difference in the vein diameter distribution in each study, we defined the proportion of patients with vein diameter <2 mm less than 30% or more than 70% as unbalanced distribution. There was no significant difference in functional maturation rates in between articles with different tourniquet using, gender ratio and vein diameter distribution (Supplementary Table S2 and Figure S1). The funnel plot (Supplementary Figure S2) showed there was no publication bias among 8 included studies, which was further confirmed by Egger's test (*P* = 0.22). According to GRADE system, the certainty of evidence was evaluated moderate to high certainty (Supplementary Figure S3). Although the included articles all belonged to observational study, the quality of evidence was upgraded due to large effect and assessing plausible confounding factors.

## Discussion

Improving the maturation rate of AVF was very important to ensure the quality of life of patients with ESKD. Several previous studies analyzed the risk factors for AVF maturation. Lannery S. Lauvao et al. (6) suggested that the vein diameter was the major predictor of fistula maturation. Crystal A Farrington et al. (24) retrospectively identified 300 patients constructing AVF in 6 years, which concluded the arterial diameter may be a predictor of AVF maturation. Race was also an important risk factor for AVF functional maturation (25). Other mentioned risk factors included body mass index (26), systolic blood pressure (27), gender, peripheral vascular disease (28), diabetes (29) etc.

We compared the baseline characteristics between maturation and non-maturation group in 5 articles with complete data. However, male patients showed better maturation outcomes than females. The result was identical with some previous studies (30). The female patients had smaller vessels than males which showed difference in vascular reactivity and distensibility (31). Coronary heart disease is a risk factor for AVF non-maturation, possibly due to the poor vascular condition of patients with coronary heart disease and the increased risk of thrombosis. In addition, radial artery diameter does not affect AVF maturation in the included articles.

Our study mainly focused on the effect of vein diameter on the maturation of AVF. Previous studies had confirmed the effect of vein diameter on the maturation of AVF. However, there was no consensus on the specific cut-off value, such as 1.5 mm (32, 33), 2 mm (17, 20) and 2.5 mm (34, 35). A systematic review performed in 2015 indicated that the optimal range of cephalic vein for maximum performance (maturation and primary patency) of RCAVF was at least 2 mm (36). The vein diameter was usually measured by preoperative color Doppler ultrasound, which can also evaluate venous stenosis, venous thrombosis, arterial diameter, arterial flow rate, arterial flow volume etc. (37). In addition, the measurement standard of venous diameter had different requirements in different studies. Some studies tied a tourniquet around the upper arm before measuring the diameter of the vein, while others not. Studies had shown that the distensibility of the vein itself may play a more important role in the maturation of AVF (38, 39).

In our study, we chose 2 mm as the grouping standard. The functional maturation rate was significantly lower in patients with vein lesser than 2 mm. Small venous diameter mean that the probability of anastomotic stenosis was higher, and it was more difficult to achieve the expected fistula diameter, flow rate and flow volume. Although the subgroup analysis indicated the tourniquet using did not influence the results, the venous distensibility played an essential role in the maturation rate of AVF, even more important than vein diameter (40). After AVFs were formed by anastomosis with arteries, the veins with good elasticity were gradually expanded under the shear stress of high-pressure arterial blood, which can quickly meet the requirements for dialysis. It was exactly the good expansibility that the veins can tolerate the increased shear stress (38). Given that there were few articles included and the specific tourniquet specific tourniquet banding position or time in different studies, the reliability of the conclusion remained to be further verified. Then we compared the primary and cumulative patency rates in 12 months postoperatively. The result indicated no significant difference between patients with different vein diameter. Hence, we reasonably believed that the long-term patency rates of AVF with vein less than 2 mm and larger than 1.35 mm (23) especially nearby 2 mm were satisfactory with the assistance of regular postoperative ultrasound and early balloon assisted maturation or secondary intervention if necessary. There were some limitations in this study. We only included 8 observational studies. None of RCTs decreased the evidence of the meta-analysis. Besides, the retrospective studies provided inadequate information for thorough comparison and analysis. Only 2 articles provided complete data in primary and cumulative patency rates and the primary assisted patency rate was not mentioned. Most articles provide maturation and patency rates at 12 months, with insufficient data on outcomes at other time points for comparison. Heterogeneity among the included studies was unavoidable by selection bias and language bias, which reduced the evidence of the results.

## Conclusion

The vein diameter is an important factor that influences the functional maturation rate of AVF. Our study has shown that when the vein diameter is less than 2 mm, the AVF functional maturation rate is negatively affected. However, this does not impact the primary and cumulative patency rates (12 months). There exists considerable feasibility in establishing AVF using veins smaller than 2 mm.

## Data Availability

The original contributions presented in the study are included in the article/**Supplementary Material**, further inquiries can be directed to the corresponding authors.

## Supplementary material

SUPPLEMENTARY FIGURE S1

(**A**) Subgroup analysis based on tourniquet using in functional maturation rate. (**B**) Subgroup analysis based on gender ratios in functional maturation rate. (**C**) Subgroup analysis based on vein diameter distribution in functional maturation rate. Events, number of fistulas with functional maturation; OR, odds ratio; CI, confidence interval.

SUPPLEMENTARY FIGURE S2

Funnel plot among 8 included studies.

SUPPLEMENTARY FIGURE S3

The certainty of evidence evaluated by GRADE system (https://www.gradepro.org/).
