# Supplementary figures and images for: The feasibility and efficiency for constructing arteriovenous fistula with <2 mm vein—a systematic review and meta-analysis
Source: Front Cardiovasc Med. 2023 Sep 18;10:1226136. doi: 10.3389/fcvm.2023.1226136 (PMC10552868; doi:10.3389/fcvm.2023.1226136)

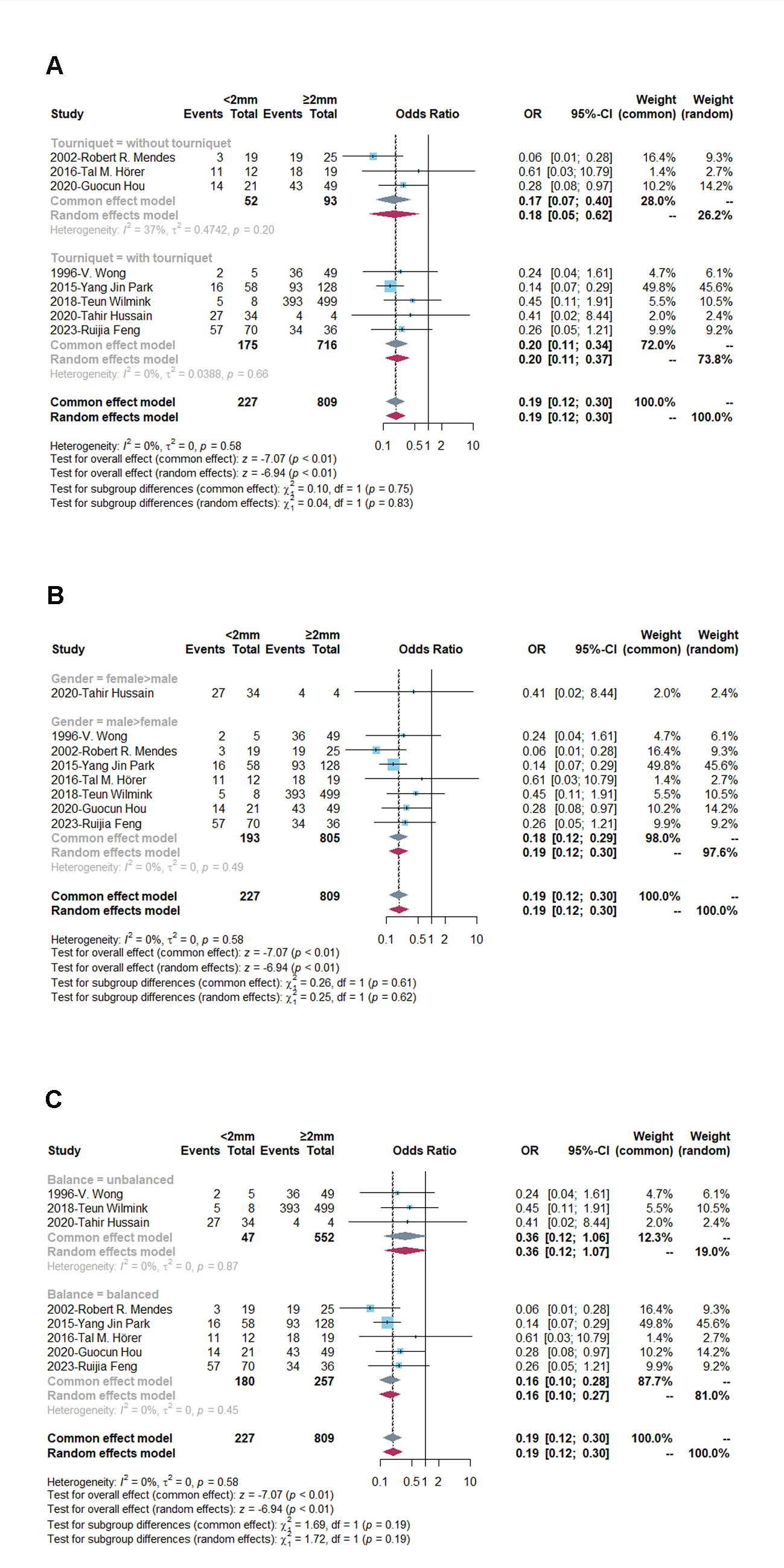

Supplement: Supplementary file 1 [file Image1.tif]

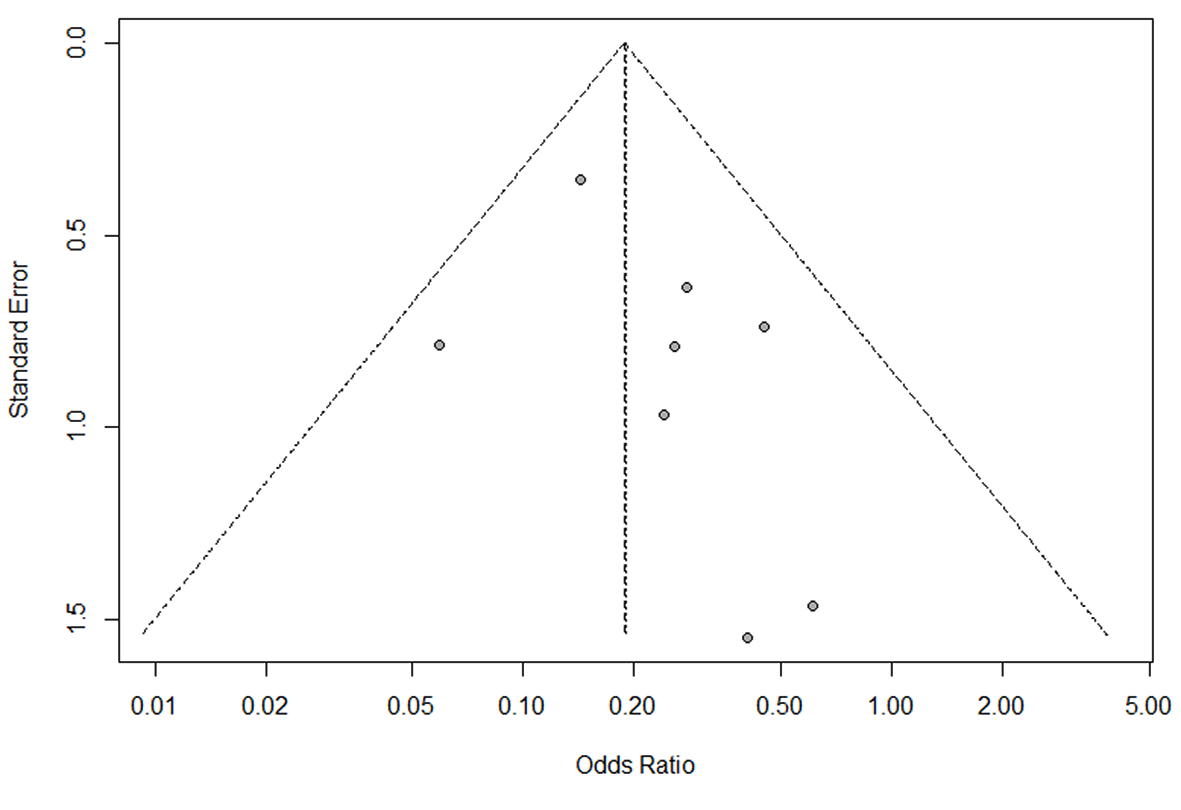

Supplement: Supplementary file 2 [file Image2.tif]

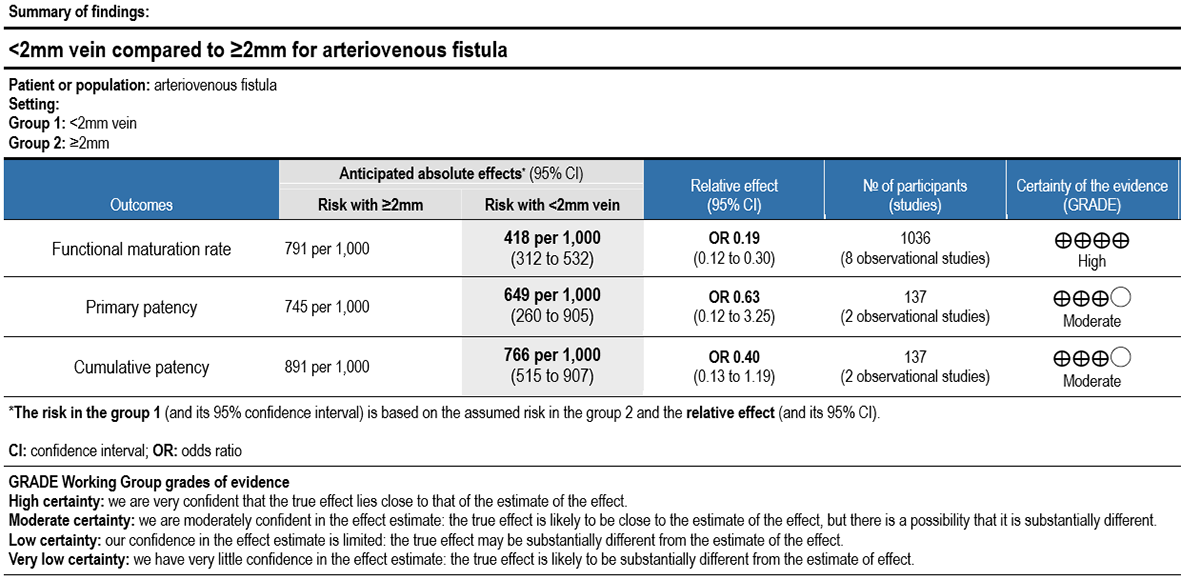

Supplement: Supplementary file 3 [file Image3.tif]
