# Supplementary material for: The feasibility and efficiency for constructing arteriovenous fistula with <2 mm vein—a systematic review and meta-analysis
Source: Front Cardiovasc Med. 2023 Sep 18;10:1226136. doi: 10.3389/fcvm.2023.1226136 (PMC10552868; doi:10.3389/fcvm.2023.1226136)
Supplement: Supplementary file 4 [file Table1.docx]

Table S1: Risk of bias assessment based on Newcastle-Ottawa Scale (NOS) for cohort study.

|  |  | V. Wong et al, 1996 | | Robert R. Mendes et al, 2002 | | Yang Jin Park et al, 2015 | | Tal M. Hörer et al, 2016 | | Teun Wilmink et al, 2018 | | Guocun Hou et al, 2020 | | Tahir Hussain et al, 2020 | | Ruijia Feng et al, 2023 |
| --- | --- | --- | --- | --- | --- | --- | --- | --- | --- | --- | --- | --- | --- | --- | --- | --- |
| Selection | 1.Representativeness of the exposed cohort | * | * | | * | | * | | * | | * | | * | | * | |
|  | 2.Selection of the non-exposed cohort | * | * | | * | | * | | * | | * | | * | | * | |
|  | 3.Ascertainment of exposure | * | * | | * | | * | | * | | * | | * | | * | |
|  | 4.Demonstration that outcome of interest was not present at start of study | * | * | | * | | * | | * | | * | | * | | * | |
| Comparability | 1.Comparability of cohorts on the basis of the design or analysis |  |  | | ** | |  | | ** | | ** | | ** | | ** | |
| Outcome | 1.Assessment of outcome | * | * | | * | | * | | * | | * | | * | | * | |
|  | 2.Was follow-up long enough for outcomes to occur |  |  | | * | | * | | * | | * | |  | | * | |
|  | 3.Adequacy of follow up of cohorts | * | * | | * | | * | | * | | * | | * | | * | |
| Total score | | 6 | 6 | | 9 | | 7 | | 9 | | 9 | | 8 | | 9 | |

A study can be awarded a maximum of one star for each numbered item within the Selection and Outcome categories. A maximum of two stars can be given for Comparability.

Table S2: Postoperative patency rates (12months) for patients with different vein diameter.

|  | Vein diameter <2mm | | | Vein diameter ≥2mm | | |
| --- | --- | --- | --- | --- | --- | --- |
|  | Total | Events | Rate | Total | Events | Rate |
| **Primary patency** |  |  |  |  |  |  |
| Tal M. Hörer et al, 2016 | 12 | 7 | 58.33% | 19 | 9 | 47.37% |
| Ruijia Feng et al, 2023 | 70 | 49 | 70.00% | 36 | 32 | 88.89% |
| **Cumulative patency** |  |  |  |  |  |  |
| Tal M. Hörer et al, 2016 | 12 | 9 | 75.00% | 19 | 15 | 78.95% |
| Ruijia Feng et al, 2023 | 70 | 57 | 81.43% | 36 | 34 | 94.44% |
